# Cyberful – Virtual Reality in der Arm- und Handrehabilitation

**DOI:** 10.1007/s00113-025-01537-2

**Published:** 2025-03-18

**Authors:** L. A. Pardo Jr., M. Markovic, I. Michelis, J. Ernst

**Affiliations:** 1 Medizinische Hochschule Hannover, Klinik für Unfallchirurgie, Carl-Neuberg-Str. 1, 30625 Hannover, Deutschland; 2 Universitätsmedizin Göttingen, Klinik für Unfallchirurgie, Orthopädie und Plastische Chirurgie, Göttingen, Deutschland; 3 Routine Health GmbH, Düsseldorf, Deutschland

**Keywords:** Amputation, Obere Extremität, Sensorische Störungen, Chronische Schmerzen, Ergotherapie, Amputation, Upper extremity, Sensory disorders, Chronic pain, Occupational therapy

## Abstract

Die Umsetzung der spezialisierten Nachbehandlung von Funktions- und Schmerzstörungen des Arms und der Hand ist eine Herausforderung. Im Rahmen eines Verbundprojekts des Bundesministeriums für Bildung und Forschung (BMBF) wurde ein Virtual-Reality(VR)-System entwickelt. Durch eine innovative, nichtinvasive, orts- und infrastrukturunabhängige Visualisierungstechnologie führt es hochspezialisierte, etablierte Therapieansätze wie geführte Bewegungsübungen, Motor Imagery und Spiegeltherapie für eine nahtlose, hochspezifische und erfolgreiche sensomotorische Rehabilitation zusammen. Neben Amputationen und Nervenverletzungen adressiert diese VR-Therapie gleichermaßen weitere traumatische sowie neurologische Funktions- und Sensibilitätsstörungen der oberen Extremität und damit verbundene Schmerzphänomene. Dieser Beitrag beleuchtet die Grundlagen des Systems, seine therapeutischen Anwendungen und das Potenzial dieser innovativen Technologie zur Verbesserung der gegenwärtigen sensomotorischen Rehabilitation und Lebensqualität von Betroffenen.

## Abstract (en)

The implementation of specialized follow-up treatment of functional disorders and associated pain of the arm and hand is a challenge. As part of a collaborative project funded by the German Federal Ministry of Education and Research (BMBF), an innovative and noninvasive visualization technology was developed that is independent of the location and infrastructure. The virtual reality (VR) system NeuroXR integrates highly specialized and established treatment approaches, such as guided movement exercises, motor imagery and mirror therapy for successful seamless and highly specific sensorimotor rehabilitation. This VR treatment addresses not only amputations and nerve injuries but also other traumatic and neurological functional and sensory disorders of the upper extremities, along with associated pain phenomena. This article explores the fundamental principles of the system, its therapeutic applications and the potential of this innovative technology to improve current sensorimotor rehabilitation practices and improve the quality of life for affected individuals.

Eine erfolgreiche sensomotorische Rehabilitation von Arm- und Handverletzungen ist herausfordernd. Die Kombination aus Beweglichkeit, Kraft und Sensorik basiert auf dem einzigartigen Zusammenspiel von Muskeln, Sehnen, Gelenken und Nerven [2, 3]. Die funktionelle Arm-Hand-Einheit ermöglicht präzise Bewegungen für eine reibungslose Durchführung alltäglicher Aktivitäten. Störungen in Funktion und Sensibilität im Arm- sowie im Schulterbereich beeinträchtigen die Lebensqualität erheblich und sind oft mit langen Krankenhausaufenthalten und Langzeitbehinderungen verbunden [1].

## Gegenwärtige Zahlen und Kosten

In der Mehrzahl der Fälle handelt es bei Eintritt der Verletzung um junge Menschen [4–8]. Daher überrascht es nicht, dass diese Verletzungen hohe (Langzeit‑)Behandlungs- und volkswirtschaftliche Kosten durch Arbeitsausfall oder Frührente generieren. Verletzungen der Handnerven (ICD-10-Code: S44, S64) führen durchschnittlich zu 147 Krankheitstagen, wobei 30 % der Betroffenen dauerhaft aus dem Berufsleben ausscheiden. Dies verursacht pro betroffener Person lebenslange medizinische und soziale Kosten in Höhe von etwa 102.000 € [7]. Frakturen der Hand (ICD-10-Code: S52-S62) resultieren in mindestens 64 Krankheitstagen, mit durchschnittlichen Gesamtkosten von 106.000 € pro Fall [9]. Noch schwerwiegender sind Verletzungen des Plexus brachialis (ICD-10-Code: S14.3, G54.0); es sind 80 % der Betroffenen dauerhaft berufsunfähig. Rund 70 % dieser Betroffenen leiden zudem an chronischen Schmerzen, die nahezu lebenslange Behandlungen erforderlich machen, was mit Kosten zwischen 35.000 und 800.000 € einhergeht [10]. Zusätzlich können selbst scheinbar geringfügige Verletzungen schwerwiegende Komplikationen wie das komplexe regionale Schmerzsyndrom (CRPS, ICD-10-Code: G90.5-.7), bei dem nur 20 % der Patienten eine vollständige Genesung erreichen, auslösen. Mehr als 50 % dieser Betroffenen kehren nie wieder in ihren Beruf zurück, wobei jährliche Behandlungskosten von etwa 6000 € entstehen [11].

Arm- und Handverletzungen verursachen hohe (Langzeit‑)Behandlungs- und volkswirtschaftliche Kosten

Die gegenwärtige sensomotorische Rehabilitation der genannten Verletzungsmustern besteht in einer phasen- und versorgungsspezifischen Physio- und Ergotherapie (z. B. instabil, belastungs- und übungsstabil). Es konnte gezeigt werden, dass aktive und visualisierte Bewegungsübungen das funktionelle Behandlungsergebnis messbar verbessern [12–14]. Zur Behandlung assoziierter Schmerzen ist besonders der Einsatz von Spiegeltherapie wirksam und anderen nichtmedikamentösen Therapiestrategien überlegen [15–19]. Der gegenwärtige Goldstandard stößt jedoch in seiner Umsetzung oft an seine Grenzen. Insbesondere der verzögerte Behandlungsbeginn durch lange Wartezeiten auf spezialisierte Therapeuten und damit verbundene zu geringe Frequenz von Behandlungen und/oder unspezifische Maßnahmen resultieren in irreversiblen Funktionseinschränkungen und chronifizierten Schmerzen [20]. Prognosen zeigen, dass die Kluft zwischen Bedarf und Verfügbarkeit noch größer wird. In Deutschland leiden 14 Mio. Menschen an chronischen Schmerzen, davon ca. 35 % aufgrund von Verletzungen oder Funktionseinschränkungen der oberen Extremität [21]. Dem stehen 56.000 und 166.000 registrierte Ergo- und Physiotherapeuten, von denen weniger als die Hälfte auf die Behandlung von Erkrankungen und Verletzungen der oberen Extremität spezialisiert sind, gegenüber [22, 23]. Während 2020 ein Anteil von 15 % der benötigten Therapieleistungen nicht gedeckt werden konnte, wird dieser Wert bis 2030 voraussichtlich auf 25 % ansteigen [20]. Diese Versorgungslücke erhöht das Risiko irreversible Funktionseinschränkungen und chronifizierte Schmerzen im Rahmen von Verletzungen oder verletzungsunabhängigen Funktionseinschränkungen der oberen Extremität und erfordert innovative Lösungen.

## Virtual-Reality-System

### Zielsetzung und Konzept

Ein vielversprechender Ansatz zur Überwindung oben genannter Lücken und Hürden ist der Einsatz von Virtual Reality (VR) als innovative Technologie zu Visualisierung, Anleitung und Dissemination wirksamer Therapiestrategien wie gezielter Bewegungsübungen, „graded motor imagery“ und Spiegeltherapie [24]. Diese Strategien zielen darauf ab, die verletzte obere Extremität individuell und spezifisch zu beüben sowie gleichermaßen die kortikale propriozeptive Repräsentation der bewegten Hand zu rekonstruieren, um den Schmerz zu reduzieren. Der Einsatz von VR-Technologie ermöglicht einen unmittelbaren Zugang zu einer hochspezifischen Therapie, die sich Gamifikation (beschreibt den Einbau spielerischer Elemente) und Nudging-Elemente („nudging“: anstoßen, anschubsen – eine Methode, die durch gezielte Anreize und Hinweise Verhaltensänderungen fördert) zunutze macht, um möglicherweise die Compliance zu erhöhen [25].

Das VR-System nutzt neue Möglichkeiten der motorischen und sensorischen Rehabilitation

Im Rahmen eines vom Bundesministerium für Bildung und Forschung (BMBF) geförderten Forschungsprojekts wurde ein VR-System entwickelt, in das die neuen Möglichkeiten der motorischen und sensorischen Rehabilitation implementiert sind. Während das System ursprünglich die Schmerztherapie nach Amputations- und Nervenverletzungen fokussierte, wurde es in einer Anschlussförderung schrittweise erweitert, um auch Bewegungstherapien zu integrieren. So ist ein flexibles, nutzerfreundliches System entstanden, das für eine Vielzahl von Indikationen geeignet ist.

Das VR-System ist sowohl für den klinischen Einsatz als auch für die Nutzung im häuslichen Umfeld konzipiert. Besonders wertvoll ist es für Patienten mit chronifizierten Verläufen, da es eine kontinuierliche, ambulante Therapie, die unabhängig von der Verfügbarkeit eines Therapeuten und Infrastrukturen durchgeführt werden kann, ermöglicht. Diese Flexibilität verbessert nicht nur die Compliance, sondern bietet auch eine effektive Lösung, die Versorgungslücke in der Rehabilitation zu schließen.

### Komponenten

#### Hardware

**Abb. 1:**
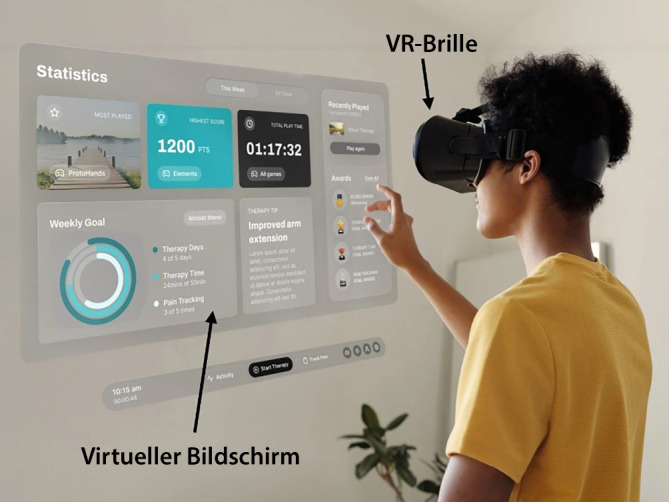
Exemplarisches Set-up des NeuroXR-Systems. Der Patient trägt eine VR-Brille, durch die Therapieanweisungen, spielerische Übungen und weitere Inhalte in einer immersiven virtuellen Umgebung dargestellt werden. Die Interaktion mit der virtuellen Welt erfolgt intuitiv über Handgesten und durch das gezielte Drücken von Knöpfen mit den Fingern

Das System verwendet die Meta Quest 3, eine VR-Brille, die über eine präzise Handtracking-Technologie verfügt. Diese erlaubt die Steuerung innerhalb der virtuellen Umgebung allein durch natürliche Hand- und Armbewegungen, ohne dass zusätzliche Controller benötigt werden (Abb. 1). Im aktuellen System haben wir das Nutzer-Feedback aus der Proof-of-concept-Studie (Publikation in Vorbereitung) in maximaler Form zur Weiterentwicklung der visuellen Therapie berücksichtigt.

### Software

Die Software des Systems ist modular aufgebaut und bietet verschiedene Ansätze zur Förderung sensorischer und motorischer Fähigkeiten:

#### ProtoHands: Förderung der Hand-Auge-Koordination und Beweglichkeit in der Akutphase.

**Abb. 2:**
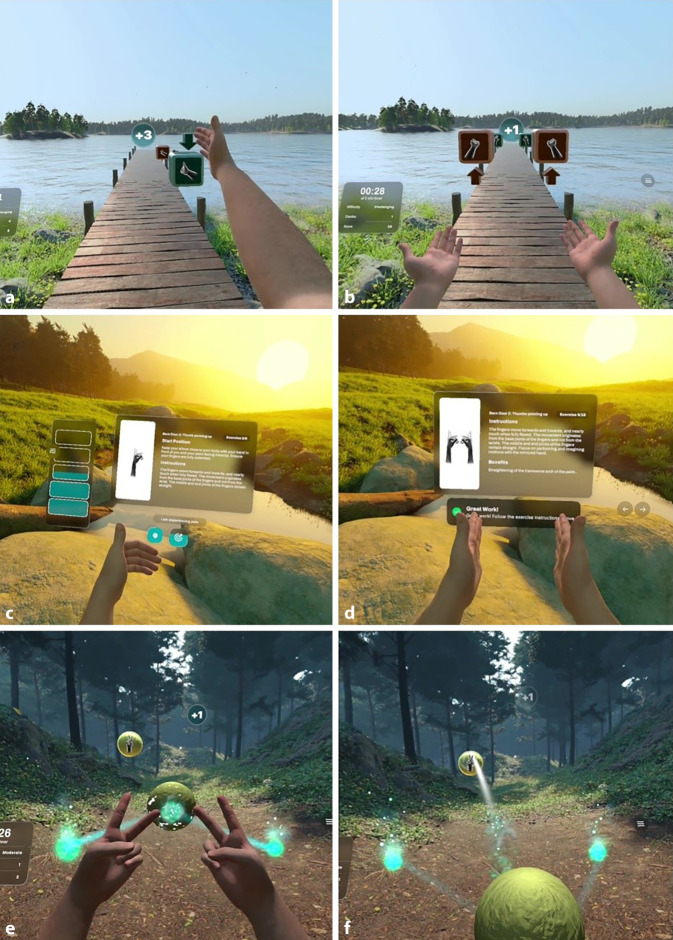
Module der NeuroXR-Virtual Reality-Therapieanwendung in verschiedenen Phasen der Rehabilitation. **a,b** Im Modul ProtoHands müssen Benützer virtuelle Kisten in eine vorgegebene Richtung schieben, wobei spezifische Handgesten ausgeführt werden. Das Modul kann sowohl ein- (**a**) als auch zweihändig (**b**) gespielt werden. **c,d** Die Bewegungstherapie umfasst eine Kalibrierung, um die Übungen individuell an den Bewegungsumfang des Patienten anzupassen (**c**), gefolgt von der eigentlichen Durchführung der Übungen (**d**). **e,f** Im Modul Elements können Patienten die Farbe eines Projektils mithilfe einer Handgeste an die des Zielobjekts anpassen (**e**). Das Projektil wird anschließend durch eine Schleuderbewegung auf das Ziel abgefeuert (**f**)

Das Modul ProtoHands ist durch populäre VR-Spiele, darunter „Beat Saber“, inspiriert und überträgt deren Konzepte in einen therapeutischen Kontext. In einer immersiven Umgebung muss der Benutzer virtuelle Kisten mithilfe spezifischer Handbewegungen in die vorgegebene Richtung schieben (Abb. 2a,b). Sowohl die Hand-Auge-Koordination als auch die Beweglichkeit des Arms werden trainiert. Das intuitive Design des Moduls sorgt für eine spielerische Erfahrung, die besonders motivierend wirkt und sich für die fortgeschrittene Rehabilitationsphase eignet, da die Bewegungsabläufe in einem größeren Bewegungsumfang der großen Gelenke erfolgen und durch die musikalische Untermalung richtig Spaß machen. In der Rehabilitation von Handverletzungen kann diese Anwendung jedoch auch schon früh eingesetzt werden, um Schulter und Arm unter Schonung des Handgelenks und der Hand zu bewegen.

​

Das Modul bietet verschiedene Schwierigkeitsgrade, die von einfachen Bewegungen auf Augenhöhe bis hin zu komplexeren Aufgaben mit variierenden Höhen reichen. Dies ermöglicht eine schrittweise Steigerung der Anforderungen, wodurch Patienten kontinuierlich gefördert werden, ohne überfordert zu werden. Die Therapie konzentriert sich insbesondere auf die Verbesserung der Beweglichkeit des Arms und die Förderung der motorischen Kontrolle.

#### Geführte Bewegungstherapie: Wiederherstellung von Feinmotorik und Geschicklichkeit in der Wiederherstellungsphase.

Das Modul Bewegungstherapie orientiert sich an klassischen physiotherapeutischen Übungen, die für die Rehabilitation der Finger‑, Hand(gelenk)- und Ellenbogenbeweglichkeit entwickelt wurden. Patienten folgen detaillierten Anleitungen, die durch visuelle und textliche Demonstrationen in der virtuellen Umgebung bereitgestellt werden (Abb. 2d). Vor Beginn der Übungen wird eine Kalibrierung durchgeführt, um Bewegungsreichweite und -umfang individuell an den Patienten anzupassen und sicherzustellen, dass die Übungen schmerzfrei ausgeführt werden können (Abb. 2c).

In der praktischen Anwendung richtet sich das Modul an Patienten, die sich in der frühen bis mittleren Rehabilitationsphase mit „kleinen Bewegungen“ befinden oder wenn postoperative Schwellungen reduziert werden sollen. Es fördert die Wiederherstellung von Feinmotorik und Geschicklichkeit durch gezielte, repetitive Bewegungen, die den Bewegungsumfang und die Präzision der Handfunktionen verbessern. Dieses Modul ist besonders wertvoll für Patienten mit Sehnenverletzungen, -frakturen, (chronischen) Schmerzen oder neurologischen Störungen, die eine intensive und zielgerichtete Therapie benötigen.

#### Elements: Präzisions- und Timing-Training als fortgeschrittene Rehabilitation.

Das Modul Elements legt den Fokus auf Präzision und unterstützt Patienten, die sich in der mittleren bis fortgeschrittenen Phase der Rehabilitation befinden. Die Therapie besteht darin, bewegliche Ziele in der virtuellen Umgebung mit Projektilen zu treffen. Der Benutzer wählt die Projektile durch spezifische Handgesten aus (Abb. 2e) und benötigt für deren Abschuss sowohl Genauigkeit als auch Timing (Abb. 2f).

Dieses Modul eignet sich besonders für die Mobilisierung der Schulter, die Förderung der Fingerbeweglichkeit und die Schulung der motorischen Kontrolle. Dank eines adaptiven Designs passt sich die Schwierigkeit der Übungen den individuellen Fähigkeiten des Patienten an, was eine stetige Herausforderung und nachhaltige Fortschritte gewährleistet. „Elements“ bietet eine vielseitige Möglichkeit, sowohl präzise Bewegungen zu trainieren als auch die Interaktion mit virtuellen Objekten zu fördern.

### Resümee

Die unmittelbare Verfügbarkeit des VR-Systems ermöglicht es, die Therapie frühzeitig zu beginnen, wodurch irreversible funktionelle Einschränkungen und chronifizierte Schmerzen vermieden werden können. Therapien regelmäßig und eigenständig durchzuführen, spielt eine zentrale Rolle bei der effizienten und nachhaltigen Wiederherstellung der Funktionalität. Die Kombination aus spielerischen Elementen (Gamification, Nudging) und personalisierten Übungen steigert die Motivation und fördert eine langfristige Therapieadhärenz.

Das VR-System ist unabhängig von Infrastrukturen und bietet die Chance auf frühen Therapiebeginn

Die Individualisierbarkeit des Systems und der unkomplizierte Einsatz im ambulanten, häuslichen Bereich bieten einen entscheidenden Vorteil für Betroffene, die durch eingeschränkte Mobilität oder fehlenden Zugang zu spezialisierten Einrichtungen benachteiligt wären. Von diesen Charakteristika der VR-Therapie könnte auch das Gesundheitssystem, u. a. durch eine Reduktion langfristiger Behandlungskosten, profitieren.

Erste klinische Untersuchungen zeigen, dass die VR-Therapie nicht nur die körperliche Funktionalität verbessert, sondern auch die Wahrnehmung des virtuellen Körpers als eigenen fördert; ein Phänomen, das als „Embodiment“ bezeichnet wird [26]. Patienten berichten von einer deutlichen Schmerzreduktion und einer Verbesserung der Mobilität nach der Anwendung des Systems.

Zusätzlich ermöglicht die Konfiguration des Systems Unabhängigkeit von Infrastrukturen und einen frühzeitigen Therapiebeginn nach einer Verletzung, was nachweislich die Rehabilitationsergebnisse verbessert. Durch die kontinuierliche Datenerfassung im System können zudem Therapiefortschritte in Echtzeit überwacht und die Therapie individuell angepasst werden.

## Fazit für die Praxis

- Die Virtual-Reality(VR)-Therapie ergänzt traditionelle Rehabilitationsansätze durch frühzeitige, personalisierte und motivierende Behandlung von Mobilitäts- und Schmerzstörungen der oberen Extremitäten. Sie kann sowohl in Kliniken als auch im ambulanten, häuslichen Umfeld effektiv eingesetzt werden:
  1. Flexibilität: Therapie ohne zeitliche und räumliche Einschränkungen, ideal für Patienten mit eingeschränkter Mobilität und/oder in infrastrukturschwachen Regionen;
  2. Motivationsförderung: gamifizierte Elemente und direktes Feedback steigern die Therapieadhärenz;
  3. individuelle Anpassung: kontinuierliche Datenerfassung ermöglicht höchst personalisierte Therapien.
- Die Integration in die ambulante Routine könnte die Versorgungsqualität erhöhen und Kosten senken, indem Komplikationen wie chronische Schmerzen und Funktionseinschränkungen reduziert werden.

## References

[1] Zeelenberg ML, Den Hartog D, Halvachizadeh S, Pape HC, Verhofstad MH, Van Lieshout EM (2022) The impact of upper-extremity injuries on polytrauma patients at a level 1 trauma center. J Shoulder Elbow Surg 31:914–922 34687916 10.1016/j.jse.2021.10.005

[2] De Putter CE, Selles RW, Haagsma JA, Polinder S, Panneman MJM, Hovius SER et al (2014) Health-related quality of life after upper extremity injuries and predictors for suboptimal outcome. Injury 45:1752–1758 25150751 10.1016/j.injury.2014.07.016

[3] Shahsavari H, Matourypour P, Ghiyasvandian S, Ghorbani A, Bakhshi F, Mahmoudi M et al (2020) Upper limb amputation; Care needs for reintegration to life: An integrative review. Int J Orthop Trauma Nurs 38:100773 32362398 10.1016/j.ijotn.2020.100773

[4] Walker-Bone K, Palmer KT, Reading I, Coggon D, Cooper C (2004) Prevalence and impact of musculoskeletal disorders of the upper limb in the general population. Arthritis Rheum 51:642–651 15334439 10.1002/art.20535

[5] Urwin M, Symmons D, Allison T et al (1998) Estimating the burden of musculoskeletal disorders in the community: the comparative prevalence of symptoms at different anatomical sites, and the relation to social deprivation. Ann Rheum Dis 57:649–655 9924205 10.1136/ard.57.11.649 PMC1752494

[6] Hashemi L, Webster BS, Clancy EA, Courtney TK (1998) Length of disability and cost of work-related musculoskeletal disorders of the upper extremity. J Occup Environ Med 40:261–269 9531097 10.1097/00043764-199803000-00008

[7] Bergmeister KD, Große-Hartlage L, Daeschler SC, Rhodius P, Böcker A, Beyersdorff M et al (2020) Acute and long-term costs of 268 peripheral nerve injuries in the upper extremity. PLoS ONE 15:e229530 32251479 10.1371/journal.pone.0229530 PMC7135060

[8] Morse TF, Dillon C, Warren N, Levenstein C, Warren A (1998) The economic and social consequences of work-related musculoskeletal disorders: the Connecticut Upper-Extremity Surveillance Project (CUSP). Int J Occup Environ Health 4:209–216 9876629 10.1179/oeh.1998.4.4.209

[9] Eisele A, Dereskewitz C, Kus S, Oberhauser C, Rudolf KD, Coenen M et al (2018) Factors affecting time off work in patients with traumatic hand injuries—A bio-psycho-social perspective. Injury 49:1822–1829 30054047 10.1016/j.injury.2018.07.012

[10] Dy CJ, Lingampalli N, Peacock K, Olsen MA, Ray WZ, Brogan DM (2020) Direct cost of surgically treated adult traumatic brachial plexus injuries. J Hand Surg Glob Online 2:77–79 32864587 10.1016/j.jhsg.2019.12.001 PMC7454232

[11] Scholz-Odermatt SM, Luthi F, Wertli MM, Brunner F (2019) Direct health care cost and work incapacity related to complex regional pain syndrome in Switzerland: a retrospective analysis from 2008 to 2015. Pain Med 20:1559–1569 30848817 10.1093/pm/pnz030

[12] Maugeri G, D’Agata V, Trovato B, Roggio F, Castorina A, Vecchio M et al (2021) The role of exercise on peripheral nerve regeneration: from animal model to clinical application. Heliyon 7:10.1016/j.heliyon.2021.e08281 PMC8571504 34765794

[13] Birinci T, Mutlu EK, Altun S (2022) The efficacy of graded motor imagery in post-traumatic stiffness of elbow: a randomized controlled trial. J Shoulder Elbow Surg 31:2147–2156 35803550 10.1016/j.jse.2022.05.031

[14] Dilek B, Ayhan C, Yagci G, Yakut Y (2018) Effectiveness of the graded motor imagery to improve hand function in patients with distal radius fracture: A randomized controlled trial. J Hand Ther 31:2–9 29122370 10.1016/j.jht.2017.09.004

[15] Seyyah M, Topuz S (2023) The effect of mirror therapy on joint movement, pain and functionality in acute upper limb burns. Burns 49:1432–1438 36754643 10.1016/j.burns.2022.11.002

[16] Bansal K, Bansal N (2023) Effect of mirror therapy on upper extremity function in a patient with humeral fracture: a case study. Phys Ther 5:108–111

[17] Fernández-Solana J, Álvarez-Pardo S, Moreno-Villanueva A, Santamaría-Peláez M, González-Bernal JJ, Vélez-Santamaría R et al (2024) Efficacy of a Rehabilitation Program Using Mirror Therapy and Cognitive Therapeutic Exercise on Upper Limb Functionality in Patients with Acute Stroke. Healthcare 12:569 38470680 10.3390/healthcare12050569 PMC10931296

[18] Muñoz-Gómez E, Aguilar-Rodríguez M, Mollá-Casanova S, Sempere-Rubio N, Inglés M, Serra-Añó P (2024) A randomized controlled trial on the effectiveness of mirror therapy in improving strength, range of movement and muscle activity, in people with carpal tunnel syndrome. J Hand Ther 10.1016/j.jht.2024.02.007 38458950

[19] Makin TR, Flor H (2020) Brain (re) organisation following amputation: Implications for phantom limb pain. Neuroimage 218:116943 32428706 10.1016/j.neuroimage.2020.116943 PMC7422832

[20] Driscoll OSW (2000) J Rehabil Res Dev 37:179–188 10850824

[21] Häuser W, Schmutzer G, Hinz A, Hilbert A, Brähler E (2013) Prävalenz chronischer Schmerzen in Deutschland. Schmerz 27:46–55 23321703 10.1007/s00482-012-1280-z

[22] Statista GmbH (2024) from. https://de.statista.com/statistik/daten/studie/520500/umfrage/anzahl-beschaeftigter-physiotherapeuten-in-deutschland/. Zugegriffen: Online

[23] Statista GmbH (2024) from. https://de.statista.com/statistik/daten/studie/520504/umfrage/anzahl-beschaeftigter-ergotherapeuten-in-deutschland/. Zugegriffen: Online

[24] Zhang J, Yang J, Xu Q, Xiao Y, Zuo L, Cai E (2024) Effectiveness of virtual reality-based rehabilitation on the upper extremity motor function of stroke patients: A protocol for systematic review and meta-analysis. PLoS ONE 19:e313296 39509415 10.1371/journal.pone.0313296 PMC11542779

[25] Auf H, Dagman J, Renström S, Chaplin J (2021) Gamification and nudging techniques for improving user engagement in mental health and well-being apps. Proc Des Soc 1:1647–1656

[26] Pozeg P, Palluel E, Ronchi R, Solcà M, Al-Khodairy AW, Jordan X et al (2017) Virtual reality improves embodiment and neuropathic pain caused by spinal cord injury. Neurology 89:1894–1903 28986411 10.1212/WNL.0000000000004585 PMC5664293

